# Early initiation of sodium‐glucose linked transporter inhibitors (SGLT‐2i) and associated metabolic and electrolyte outcomes in diabetic kidney transplant recipients

**DOI:** 10.1002/edm2.185

**Published:** 2020-09-13

**Authors:** Chelsey Chenxi Song, Andrew Brown, Ryan Winstead, Idris Yakubu, Moses Demehin, Dhiren Kumar, Gaurav Gupta

**Affiliations:** ^1^ Hume‐Lee Transplant Center Virginia Commonwealth University Health Richmond VA USA

## Abstract

There is a paucity of data on the use of SGLT2 inhibitors on outcomes in kidney transplant recipients. There may be concern in initiating these agents, especially within the first year post‐transplant when renal function is more labile and immunosuppression more intense, due to a presumed high risk of urinary infections and acute kidney injury. This is a retrospective study on 50 kidney transplant recipients, half of whom were started on therapy within the first year of transplant. Over a follow‐up period of 6 months, overall patients had a statistically significant improvement in weight by −2.95 kg [SD 3.54, *P* = <.0001 (CI: 3.53, 1.50)] as well as hypomagnesemia 0.13 [SD 1.73, *P *= .0004 (CI: 0.06, 0.20)]. Overall insulin usage declined by −3.7 units [SD 22.8, *P* = .17]. 14% of patients had at least one urinary tract infection although this rate is not different (~20%) than that reported historically in this high‐risk population.

## Graphical abstract

Sodium‐glucose Linked Transporter Inhibitor (SGLT‐2i) are widely used in the nontransplant population for its multitude of benefits including cardiovascular as well as renal projective effects. However its efficacy and safety in the solid organ transplant population is largely unknown, particularly in the kidney transplant population. This study aims to evaluate the safety and well as electrolyte benefits of SGLT‐2i in this particularly vulnerable population.

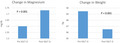

Post‐transplant diabetes (PTDM) is commonly associated with increased morbidity and mortality in kidney transplantation (KT) recipients. Electrolyte instability such as hypomagnesemia has been associated with increased risk in the development of new‐onset PTDM, worsening cardiovascular outcomes and insulin resistance post‐KT.^1^, ^2^, ^3^ The use of sodium‐glucose linked transporter inhibitors (SGLT‐2i) in nontransplant diabetic patients has demonstrated reduced cardiovascular mortality, delayed chronic kidney disease (CKD) progression and correction of hypomagnesemia.^4^, ^5^ Currently, there are data on the use of SGLT‐2i in KT recipients within the first year post‐transplant when renal function may be lower and immunosuppression more intense. These factors may increase the vulnerability of KT recipients to SGLT‐2i‐associated adverse events such urinary tract infections (UTIs), diabetic ketoacidosis (DKA) and prerenal acute kidney injury (AKI). However, early initiation of SGLT‐2i within the first year post‐KT may provide significant benefits not only in glycemic control and metabolic side effects, but also in long‐term hypomagnesemia associated with chronic calcineurin inhibitor utilization. Proper management of these complications early on post‐transplant is needed to curb the progression to chronic disease and deleterious graft outcomes. This study aims to evaluate the metabolic, electrolyte and safety outcomes of early SGLT‐2i utilization in KT recipients.

This was a single centre, retrospective, institutional review board approved study conducted in adult KT recipients who met our SGLT‐2i initiation criteria. Patients were eligible if they had type II diabetes (pre‐existing type 2 diabetes or PTDM); absence of AKI ≤30 days prior to initiation of therapy; freedom from any UTIs 6 months prior to therapy initiation; and an estimated glomerular filtration rate (eGFR) ≥30 mL/min at the time of initiation. Primary outcomes were changes in weight, insulin dosage, haemoglobin A1C (HgbA1C), magnesium concentration and safety outcomes including treated UTIs, DKA, amputations and hospitalizations due to AKI. The choice of SGLT‐2i was chosen based on patient's individual insurance coverage.

Selected outcomes are summarized in Table 1. Of the 62 patients evaluated, 50 met inclusion criteria. Thirty‐nine (78%) received deceased donor KT, and thirty‐three (66%) were males. The median time to drug initiation was 319 days (IQR 112, 696) from transplant, twenty (40%) of whom were started within 200 days post‐transplant. The mean eGFR at the time of initiation was 66.7 ± 20.6 mL/min with 7 (14%) patients initiating therapy with an eGFR between 30 and 45 mL/min. There were no changes in renal function 3 and 6 months post‐therapy. Forty‐three (86%) patients received empagliflozin, 6 (12%) canagliflozin and 1 (2%) dapagliflozin. A significant improvement in weight by −2.95 kg [(SD 3.54, *P *= <.0001 (CI: 3.53, 1.50)] and increase in magnesium concentration by 0.13 [(SD 1.73, *P* = .0004 (CI: 0.06, 0.20)] were seen within a mean follow‐up of 101 days (Figure 1). Overall insulin requirements also decreased by −3.7 units, however not statistically significant (SD: 22.8 *P* = .17). None of the patients experienced DKA, amputations or AKI episodes. Seven (14%) developed UTIs on average 69.4 days after drug initiation. Therapy was discontinued in 9 patients: 5 (10%) due to UTIs, 1 (2%) developed a genital yeast infection, 1 (2%) due to native disease recurrence, 1 (2%) due to resolution of PTDM and 1 (2%) due to physician preference.

**Table 1:** Results

	N = 50	*P‐*value
Mean Age (SD)	57.03 (13.14)	
Male, n (%)	33 (66)	
DDKT, n (%)	39 (78)	
Concomitant diabetic therapy, n(%)		
Metformin	32 (64)	
GLP‐1	5 (10)	
DPP‐4	12 (24)	
Sulfonylurea	1 (2)	
Insulin	42 (84)	
Diabetes Type		
Pre‐existing Type 2 Diabetes	40 (80)	
PTDM	10 (20)	
Immunosuppression Regimen, n(%)		
Tacrolimus	45 (90)	
Mycophenolate mofetil	47 (94)	
Prednisone	49 (98)	
Time from Transplant to Drug initiation (Median, IQR)	319.5 d (122, 696)	
Started within 200 d n (%)	20 (40)	
Started within 365 d n (%)	30 (60)	
eGFR at Drug Initiation (Mean, SD)	66.7 mL/min	
30‐45 mL/min n(%)	7 (14)	
Change in eGFR (Median, IQR) postinitiation		
3 mo (IQR)	−1 mL/min (−7.5, 7)	*P* = .8831
6 mo (IQR)	1 mL/min (−8,16)	*P* = .1478
Change in HgbA1C (Mean, SD)	−0.53% (1.79)	*P* = .1189
Treated UTI (%)	7 (14)	
Change in Insulin Requirement (Mean, SD)	−3.7 units (22.8)	*P* = .17

Abbreviations: DDKT, deceased donor kidney transplant; DPP‐4, dipeptidyl peptidase 4 inhibitors; eGFR, estimated glomerular filtration rate (mL/min/1.73m2); GLP‐1, glucagon‐like peptide‐1; HgbA1c, haemoglobin A1c; IQR, interquartile range; SD, standard deviation; UTI, urinary tract infection.

^a^ Excluding patients already on magnesium repletion therapy.

**Figure 1:**
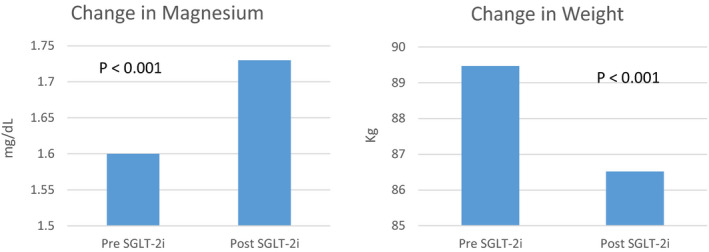
Changes in magnesium and patient weight

Despite the nonsignificant short‐term impact on diabetic management, our study illustrates that SGLT‐2i can be used safely in the management of PTDM within KT recipients. Thereby increasing the oral antidiabetic treatment arsenal, as diet and oral medications have shown to be superior in obtaining glycemic control as compared to subcutaneous insulin among KT patients.^6^ The small but statistically significant increment in magnesium concentrations might also provide benefit in KT recipients who experience chronic hypomagnesemia and decrease the progression of cardiovascular related outcomes.^1^, ^2^, ^3^, ^7^ Our incidence of adverse events particularly UTIs was comparable or even lower than those previously reported.^8^, ^9^, ^10^ Some limitations to our study include the single‐centre retrospective study design where results cannot be truly interpreted for causality and data collection was at the mercy of clinic visit documentations. Future randomized research is needed to further validate the results.

Overall, the addition of SGLT‐2i in select KT patients could provide benefit to common metabolic complications and electrolyte abnormalities such as weight gain and chronic hypomagnesaemia from prolonged immunosuppression exposure.

## CONFLICTS OF INTEREST

Dr Gaurav Gupta has served on the Scientific Advisory Board of Relypsa. The rest of the authors have no conflicts of interest or financial ties to disclose.

## **AUTHOR** **CONTRIBUTION**

Chelsey Chenxi Song, PharmD, participated in research design, participated in the writing of the paper, and participated in the performance of the research. Andrew Brown, PharmD, Idris Yakubu, PharmD, and Moses Demehin, PharmD, participated in data collection. Ryan Winstead, PharmD, participated in data collection and participated in data analysis. Dhiren Kumar, MD, participated in research design. Gaurav Gupta, MD, participated in research design and participated in writing and editing of the paper.

## Data Availability

The data that support the findings of this study are available on request from the corresponding author. The data are not publicly available due to privacy or ethical restrictions.
